# Development of a clinical prediction model using volatile organic compounds as breath biomarkers in squamous oesophageal neoplasms: ViSON, protocol for a multicentre case–control study in UK NHS hospitals

**DOI:** 10.1136/bmjopen-2025-112493

**Published:** 2026-09-28

**Authors:** Sameera Sharma, James Murray, Aaron Parker, Sara Jamel, Stefan Antonowicz, Adele Phillips, Elaine Bailey, Lyn Fox, Christine Plant, Valerio Converso, James K Ellis, Somnath Mukherjee, Bhaskar Kumar, Terence Lo, Shajahan Wahed, Alia Alchawaf, Andrew R Moore, Alex Boddy, Martin Scott-Brown, Betsan Thomas, Tarig Abdelrahman, Philip H Pucher, Ahmed El-Sharkawy, Samantha Forner, John Wayman, Ahmed Elshaer, David Chan, Stephen Mcnally, Russell Petty, Pradeep Patil, Neel Bhuva, Jambulingam Periyathambi, Lubna Bhatt, Ravinder Vohra, Linda Sharples, George B Hanna, ViSON Collaborators, Suresh Natarajan

**Affiliations:** 1 Department of Surgery and Cancer, Faculty of Medicine, Imperial College London, London, UK; 2 Department of Medical Statistics, London School of Hygiene & Tropical Medicine, London, UK; 3 Patient and Public Representatives, in Collaboration with Oeophageal Patients Association, London, UK; 4 Patient and Public Representatives, in Collaboration with Oxfordshire Oesophagus and Stomach Organisation, Oxford, UK; 5 Oxford University Hospitals NHS Foundation Trust, Oxford, UK; 6 Norfolk and Norwich University Hospitals NHS Foundation Trust, Norwich, UK; 7 Hull University Teaching Hospitals NHS Trust, Hull, UK; 8 The Newcastle Upon Tyne Hospitals NHS Foundation Trust, Newcastle Upon Tyne, UK; 9 The Clatterbridge Cancer Centre NHS Foundation Trust, Liverpool, UK; 10 Liverpool University Hospitals NHS Foundation Trust, Liverpool, UK; 11 University Hospitals of Leicester NHS Foundation Trust, Leicester, UK; 12 University Hospitals Coventry and Warwickshire NHS Trust, Coventry, UK; 13 Velindre University NHS Trust, Cardiff, UK; 14 Department of Surgery, Cardiff and Vale University Health Board, Cardiff, UK; 15 Department of Surgery, Portsmouth University Hospitals NHS Trust, Portsmouth, UK; 16 Department of Surgery, University Hospitals of Derby and Burton NHS Foundation Trust, Derby, UK; 17 Department of Oncology, Maidstone and Tunbridge Wells NHS Trust, Maidstone, UK; 18 Department of Surgery, North Cumbria Integrated Care NHS Foundation Trust, Carlisle, UK; 19 Department of Surgery, Bradford Teaching Hospitals NHS Foundation Trust, Bradford, UK; 20 Department of Surgery, University Hospitals Plymouth NHS Trust, Plymouth, UK; 21 Department of Surgery, NHS Highland, Inverness, UK; 22 Department of Oncology, NHS Tayside, Dundee, UK; 23 Department of Surgery, NHS Tayside, Dundee, UK; 24 Department of Oncology, East and North Hertfordshire NHS Trust, Northwood, UK; 25 Department of Surgery, Bedfordshire Hospitals NHS Foundation Trust, Luton, UK; 26 Department of Oncology, The Christie NHS Foundation Trust, Manchester, UK; 27 Department of Surgery, Nottingham University Hospitals NHS Trust, Nottingham, UK; 28 Department of Surgery and Cancer, Imperial College London, London, UK

**Keywords:** Early Detection of Cancer, SURGERY, Gastrointestinal tumours, Oesophageal disease, Case-Control Studies

## Abstract

**Abstract:**

**Introduction:**

Oesophageal squamous cell carcinoma (OSCC) accounts for more than 85% of the global burden of oesophageal cancer, resulting in 500 000 deaths annually. In the UK, over two-thirds of OSCC cases are diagnosed at an advanced stage, with a 5-year overall survival of less than 20%. Breath-based triage tests are feasible, acceptable and show proof of concept for earlier cancer detection.

**Methods and analysis:**

The Volatile Organic Compounds as Breath Biomarkers in Squamous Oesophageal Neoplasms (ViSON) study is a multicentre case–control study powered to estimate breath test performance with 90% sensitivity and 80% specificity. A total of 518 participants (259 with OSCC and 259 non-cancer controls) will be recruited via a network of participating National Health Service trusts. Participants will provide exhaled breath samples, which will be stored on Thermal Desorption tubes and transported to the central laboratory for processing and analysis using gas chromatography-mass spectrometry. A clinical prediction model will be developed using logistic regression with cross-validation and bootstrap methods to mitigate overfitting. Model performance will be evaluated using discrimination, calibration and accuracy. Decision curve analysis will assess clinical utility.

**Ethics and dissemination:**

The ViSON study received ethical approval from the London – Riverside Research Ethics Committee (23/PR/1300) from the Health Research Authority (HRA) and the Health and Care Research Wales (HCRW) to conduct study procedures in England and Wales. A Scotland-wide review was approved on 24 January 2024. The results of the study will be presented at national and international conferences, and a lay summary will be developed with the patient and public involvement representatives and shared using the online website, social media accounts and charity newsletters.

**Trial registration number:**

NCT06169163.

**STRENGTHS AND LIMITATIONS OF THIS STUDY**

- Given the low prevalence of oesophageal squamous cell carcinoma in the UK, the robust statistical design will efficiently estimate sensitivity and specificity of this non-invasive and highly acceptable breath test.
- The method of sampling and analysis used in this study is aligned with the International Standardisation Organisation, ensuring comparability and reproducibility of patient samples, which will be processed and analysed at a central laboratory.
- The clinical prediction model derived from this study may not be directly generalisable to the target population without recalibration to account for differences in disease prevalence.
- An intrastudy validation cohort will not be possible, and a separate narrow and broad validation study will need to be conducted.
- If required, recruitment will be expanded to ensure that the target number of patients with usable samples and confirmed diagnoses is reached.

## Introduction

### Oesophageal squamous cell carcinoma

 Oesophageal cancer (OC) is the eighth most common cancer worldwide with more than 600 000 new cases every year, of which 85% are oesophageal squamous cell carcinoma (OSCC).^1^ In the UK, there are nearly 2000 cases of OSCC every year.^2^

Typically, OSCC progression occurs through an established sequence of hyperplasia, dysplasia and carcinoma.^3^ Each stage is histologically recognisable using the reference diagnostic test: oesophago-gastro-duodenoscopy (OGD) and biopsy, coupled with chromoendoscopy and immunohistochemical staining.^4^

### Diagnosis and survival

According to the National Oesophago-Gastric Cancer Audit 2022, over two-thirds of patients with OSCC are diagnosed at an advanced stage.^1^ This diagnostic delay is multifactorial since early symptoms are non-specific and mimic benign conditions.^4^ ‘Alarm’ features that initiate investigation signal advanced disease,^5^ and the main route to diagnosis is the National Institute for Health and Care Excellence (NICE) NG12 ‘suspected cancer’ referral pathway, which focuses on alarm symptoms and older age, potentially limiting its ability to detect OSCC at an early stage.^6^

While over 70% of OSCC patients are referred by their general practitioners (GPs)^2^ and diagnosed via the NICE NG12 ‘suspected cancer’ pathway, approximately 93% of patients referred for an urgent endoscopy under this pathway do not have cancer, resulting in significant strain on resources, contributing to diagnostic delays.^7 8^

In the UK, the current incidence of OSCC by stage is 3% (stages 0–I), 25% (stage II), 39% (stage III) and 33% (stage IV). The stage of OSCC at diagnosis determines the treatment intent (curative vs non-curative) and is a key prognostic factor. Currently, due to advanced stage at diagnosis, fewer than 40% of patients receive treatment with curative intent. The 5-year overall survival exceeds 90% for patients diagnosed at an early stage (0–I), remains around 60% for locally advanced stages (II–III) and is less than 20% for advanced disease (IV).^2^

This highlights a clear unmet clinical need for a more reliable triage test to guide GPs in making endoscopy referrals and facilitate earlier detection of OSCC.

### Literature review: breath biomarkers in OSCC

In the UK, breath-biomarker research in oesophageal adenocarcinoma has transitioned from early profiling studies to multicentre validation trials with robust statistical power.^5 6^ However, there have been no published studies evaluating volatile organic compounds (VOCs) in breath for detection of OSCC. For the proposed breath test to reach its potential as a ‘triage test’ for OC, comparable efforts in OSCC are necessary.

Globally, several phase I discovery studies have provided proof of concept for the role of breath VOCs in OSCC and these studies are summarised in online supplemental table 1. As highlighted in the individual study review, the current evidence base is limited by predominantly case–control designs with non-representative control populations, leading to potential selection and spectrum bias. Studies are also largely conducted in East Asian populations, limiting generalisability and are often small with limited use of formal power calculations. There is further variability in breath sampling protocols, and substantial heterogeneity in analytical approaches, including the use of both targeted and untargeted analyses and a range of platforms [eg., gas chromatography–mass spectrometry (GC–MS), proton transfer reaction–mass spectrometry (PTR-MS), high-pressure photon ionization time-of-flight mass spectrometry (HPPI-TOF-MS), and solid-phase microextraction (SPME)-based methods], which may affect comparability and reproducibility.^9–14^

### Proposed solution: breath test

The Methodological Approaches towards a Gastrointestinal Cancer Breath Test (MAGIC) study demonstrated that breath testing has the ideal characteristics for a triage test: it is non-invasive, clinically feasible and highly acceptable in a demographically and clinically representative population, with more than 99% of participants reporting that the test was easy to perform and that they would be willing to repeat it in the future.^15^ Given the central role of GPs in cancer detection, the proposed test will benefit patients that present to primary care with non-specific symptoms but who do not meet referral criteria under the NICE NG12 pathway. In this context, breath testing could help guide appropriate referral for the reference diagnostic test (OGD).

The breath test relies on the detection of VOCs, carbon-based molecules produced during cellular metabolism. Owing to their volatility, these compounds are released in the circulation and are subsequently exhaled in breath, from where they can be captured, concentrated and analysed to identify cancer-specific signatures.^16^

### Objectives

The primary objective of the ViSON (Volatile Organic Compounds as Breath Biomarkers in Squamous Oesophageal Neoplasms) study is to develop a clinical prediction model (CPM) for estimating OSCC risk, based on comparative analysis of exhaled VOC profiles in OSCC patients and non-cancer controls. A secondary objective is to assess the predictive value of patient and clinical characteristics in addition to the VOCs.

## Methods

### Study design

The ViSON study is a UK-based, multicentre case–control study with prospective data collection, which adopts a two-gate design. Cancer cases will be recruited from patients with histopathologically confirmed OSCC, while controls will be recruited from patients undergoing endoscopy either routinely or via the ‘suspected cancer’ pathway. The study is sponsored by Imperial College London and has obtained National Institute of Health and Care Research Clinical Research Network portfolio adoption to support study recruitment across sites.

### Study setting

The recruitment timeline is between December 2023 and November 2026. The study is open to all National Health Service (NHS) trusts and 23 sites are actively participating (online supplemental material).

### Study groups and selection criteria

Participants aged over 18 years who meet either of the following inclusion criteria will be eligible for enrolment.

Inclusion criteria

- OSCC group: Participants with a confirmed histopathological diagnosis of OSCC who are treatment naive. (Treatment includes chemotherapy, radiotherapy, immunotherapy, surgical or endoscopic resection (excluding diagnostic biopsy) or any combination thereof).
- Control group: Participants who have undergone (within the past 12 months) or are undergoing an OGD as part of their investigation for upper gastrointestinal (GI) symptoms and are found to have either: (1) a normal upper GI tract or (2) benign upper GI disease.

Exclusion criteria

- Participants in the OSCC group who have had treatment for the current diagnosis.
- Participants in either group will be excluded if any of the following apply:
  - Personal history of invasive cancer or haematological malignancy diagnosed within the past 3 years (Note: non-melanoma skin cancer and prostate cancer patients whose only treatment is active surveillance are not excluded).
  - Non-squamous cell OC.
  - Barrett’s oesophagus (with or without dysplasia).
  - Squamous dysplasia without invasive cancer.
  - Previous oesophageal or gastric resection.
  - A lack of capacity or inability to provide written consent.
  - Pregnancy.

#### Rationale for choice of controls

To minimise false negative cases and ensure comparability, participants in the control group must have undergone an endoscopy within the past 12 months or be scheduled for one within 12 months of recruitment. Controls will not be individually matched to OSCC cases on factors such as alcohol consumption or smoking. Instead, their potential confounding will be assessed through statistical modelling and subgroup analyses.

### Sources of bias and mitigation strategies

The following sources of systematic bias have been identified, with strategies incorporated into the study design and analysis to mitigate their effects.

Selection bias will be minimised through consecutive sampling of all eligible participants during the study period. Participants will be identified systematically by members of the direct care team via upper GI multidisciplinary team (MDT) meetings, upper GI endoscopy lists and outpatient clinic lists. OSCC cases will be identified primarily through MDT meetings, while control participants will be recruited from upper GI endoscopy and outpatient clinic lists to ensure symptom-matched selection.

While mitigating information bias is challenging for the OSCC group, controls will primarily be recruited before endoscopy to reduce the likelihood that knowledge of their diagnosis influences recall. Measurement bias will be minimised through adherence to standardised, validated laboratory protocols and rigorous quality control measures during breath collection and analysis. All breath samples will be pseudonymised at the point of collection. Subsequent handling and analysis will be conducted with the analytical and statistical teams blinded to the outcome of the reference standard test, and therefore to case–control status.

Finally, known OSCC risk factors—including smoking history, alcohol consumption and chemical and thermal injury to the oesophagus—will not be matched across groups but will be adjusted for in subgroup and multivariable regression analyses to isolate the independent association between VOCs and OSCC.

### Sample size calculation

The sample size is powered to estimate the diagnostic accuracy with 95% CI. Assuming 90% sensitivity and 80% specificity, 139 OSCC and 246 non-cancer controls with complete data are required.^17^ To ensure sufficient power if sensitivity is lower than expected with 85% specificity, we aim for 492 participants (246 in each group) with complete data, with an inflation to 518 (259 per group) to accommodate 5% sample loss. This includes losses due to ineligibility following consent and sample exclusion during quality control procedures (eg, subthreshold detection of internal standards). Recruitment will continue if the sample loss rate exceeds 5%.

For clinical prediction model development, we followed guidance from Riley *et al* on sample size requirements.^18^ Assuming 15 predictors, a model R^2^ of 0.2, and 50% outcome prevalence, a sample size of 492 is sufficient to estimate the mean absolute prediction error <7%, acceptable model shrinkage of 12% and precisely estimate the C-statistic with SE <2%.^19^ As VOCs are often highly correlated, the number of predictors is uncertain; combined with the rarity of OSCC and use of a case–control design, these performance measures are considered reasonable.

### ViSON study procedures

Figure 1 illustrates the study procedures for the ViSON study.

**Figure 1:**
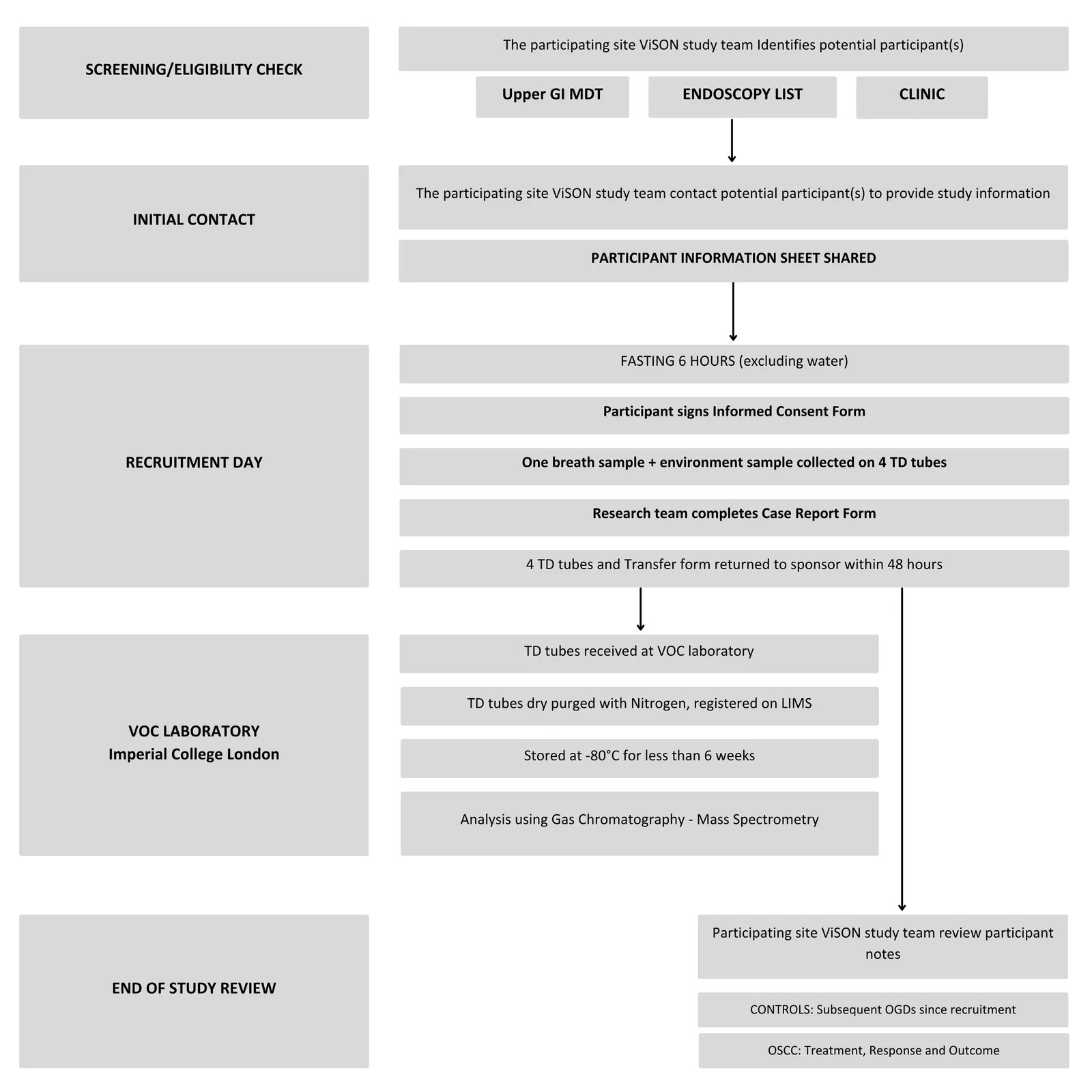
ViSON study procedures. GI, gastrointestinal; LIMS, laboratory information management system; MDT, multidisciplinary team; OGD, oesophago-gastro-duodenoscopy; OSCC, oesophageal squamous cell carcinoma; TD, thermal desorption; ViSON, Volatile Organic Compounds as Breath Biomarkers in Squamous Oesophageal Neoplasms; VOC, volatile organic compounds.

#### Participant selection

Between December 2023 and November 2026, all eligible participants will be identified consecutively by members of the direct care team at participating NHS sites via the upper GI MDT meetings (OSCC) and the upper GI endoscopy lists or outpatient clinic lists (controls). All eligible participants will be approached regarding participation in the ViSON study. If interested, a participant information sheet will be shared, and participants will be given as much time as is feasible to make an informed decision on whether to take part in the study. Where possible, the recruitment visits will be aligned with planned hospital appointments, investigations or procedures.

#### Breath collection and storage

For participants in the OSCC group, the breath test will be performed after the reference standard test and histopathological confirmation. For control participants, the breath test may be performed either before or after the reference standard test, with final eligibility confirmed following the test. Participants who do not meet the eligibility criteria will be withdrawn, and in the rare event that they meet the OSCC criteria, will be transferred to the OSCC group. All breath samples will be pseudonymised at the point of collection.

On the day of the research visit, participants must fast for 6 hours (except for water) prior to breath sampling. They will be asked to sign a written informed consent, provide two breath samples and complete a case report form (CRF).

To deliver a breath sample, participants will rinse their mouth with water and exhale into the mouthpiece of a single-use breath collection bag (Nalophan, 2 L) connected to two thermal desorption (TD) tubes (Markes International, Llantrisant, UK), which in turn are connected to a Gas Sampling Pump (GaSP). Once the breath collection bag is inflated (2 L), the mouthpiece is sealed, and the GaSP draws in the breath sample at a rate of 200 mL/min over 240 s. While passing through the TD tubes, the breath VOCs are trapped onto the sorbent material lining the tubes. After 240 s, the TD tubes are disconnected from the GaSP and recapped with long-term storage caps. The process is repeated to collect an additional blank (room air) sample using the same method as above, but without connecting the breath collection bag. For participants undergoing surgery or a procedure, breath samples must be collected in advance, prior to administration of sedation, anaesthetic or other intravenous pharmacological agents.

For each participant, four TD tubes will be utilised and transported to the central laboratory in custom-designed, magnetised boxes under ambient temperature conditions to limit contamination.

In preparation for storage, the tubes will be uncapped and dry purged using the TC20 (SERTCR-5029, SepSolve Analytical) which circulates dry nitrogen gas through the tubes to remove water vapour. Once dry purged, the tubes will be logged on to the Laboratory Information Management System (LIMS) and stored at −80°C for a maximum of 3 months before being processed.

#### VOC analysis

Breath and room air samples will be analysed at the central laboratory in accordance with established Standard Operating Procedures. Volatolomic profiling will be undertaken using TD-GC-ToF-MS instruments (Markes TD100-XR—Agilent 6890—BenchTOF Select, SepSolve, UK) equipped with mid-polar columns. Once the TD tubes have been analysed using mid-polar GC-ToF-MS, VOCs will be recollected and analysed further using a two-dimensional TD-GC/GC-ToF-MS (Markes Centri—Agilent 7890—Sepsolve modulator—BenchTOF Select-eV, Sepsolve, UK) for robust structural identification of VOCs.

The GC-MS instruments will be quality controlled before every run by analysing TD tubes loaded with known concentrations of selected VOCs (internal standards) and assessing reproducibility, mass resolution, peak area, shape and retention time. The acquired spectra will be compared against the National Institute of Standards and Technology mass spectral library by matching mass spectra-retention indices and authentic standards for chemical verification of compounds. Data will be extracted, preprocessed and quality controlled using ChromSpace (Markes, UK) and in-house scripts. Scientists analysing the GC-MS data will have access to pseudo-anonymised data.

### Statistical analysis

Statistical analyses will follow Transparent Reporting of a multivariable prediction model for Individual Prognosis or Diagnosis (TRIPOD) guidelines. A TRIPOD checklist for prediction model development has been completed and submitted alongside this study protocol.^20^ Baseline characteristics will be summarised by the study group, and the number of participants, samples collected and samples processed will be reported. Missing data patterns will be explored and reported for each variable.

Relationships between individual VOCs and OSCC will be initially explored using univariable logistic regression. Given the high dimensionality of VOC data, a screening step will identify the top (approximately) 50 VOCs for model development. This applies moderated t-statistics with empirical Bayes variance shrinkage and false discovery rate correction to rank VOCs by their univariable association with OSCC status.^21^ Variable selection for model development will use penalised regression methods, including the least absolute shrinkage and selection operator and elastic net regression, which are suitable for handling correlated predictors. Patient factors (eg, age, sex, smoking history) will be assessed for additional predictive value based on statistical or clinical relevance.^22 23^

A logistic regression-based CPM will be developed using nested cross-validation for model tuning and bootstrap resampling to assess optimism and identify stable predictors. Model performance will be evaluated in terms of discrimination [area under the receiver operating characteristic (ROC) curve], calibration (via calibration plots) and accuracy (eg, mean absolute prediction error and Brier score). Final model coefficients will be presented with ORs and 95% CIs.

The case–control design artificially sets disease prevalence at 50%, and clinical applications will require recalibration of predicted probabilities of the CPM to target population prevalence using published estimates of OSCC in relevant clinical settings. Thresholds for classification will prioritise high sensitivity (>90%) to minimise false negatives. ^24^

Decision curve analyses will be conducted to evaluate the net benefit of the CPM across a range of risk thresholds.^25^ This will allow comparison of the breath test’s utility against standard strategies (ie, referring all or no patients) and will help identify the threshold at which the benefits of detection outweigh the relative costs of false positives or missed cancers. These analyses will inform the potential role of the breath test in clinical practice and support future discussions surrounding implementation.

The final ViSON model will require external validation in independent cohorts, which may require international recruitment.

### Future work

This work forms part of a staged validation and clinical effectiveness programme. A narrow external validation in a similar UK population using a two-gate approach will be followed by a prospective one-gate validation study in the intended-use primary care population of patients referred for upper GI endoscopy, in which the breath test will be applied prior to the reference standard.

An international, multicentre study in regions with a high prevalence of OSCC, including Asia, East Africa and South America, will evaluate model performance across geographically and demographically diverse populations and will explore potential variations in breath-based metabolic signatures between populations. The effectiveness of the test in primary care decision-making, referral patterns in the cancer diagnostic pathway, costs, healthcare system resource utilisation, health inequality and clinical outcomes will need to be measured in a large-scale effectiveness and implementation study.

### Patient and public involvement and engagement

Patients and members of the public from the Oesophageal Patients Association (OPA) and Oxfordshire Oesophageal and Stomach Organisation (OOSO) were approached through charity newsletters to develop the research question and review the English summary and patient-facing documents.

Four members were appointed to the public advisory group (PAG) and are actively involved in the trial steering committee meetings, maintaining the social media webpage and developing patient awareness videos.

On completion of the study, a lay summary will be developed with members of the PAG and disseminated through presentations, publications and charity newsletters.

## Ethics and dissemination

### The research ethics committee review

The ViSON study received ethical approval (23/PR/1300) from the Health Research Authority (HRA) and the Health and Care Research Wales (HCRW) to conduct study procedures in England and Wales. A Scotland-wide review was approved on 24 January 2024. The study requires confirmation of the capacity and capability from each participating NHS Trust before accepting participants and before any research activity is carried out. The study will be conducted in accordance with the recommendations for physicians involved in research on human subjects adopted by the 18th World Medical Assembly, Helsinki 1964, and later revisions.

Any modifications to the protocol that may impact the study conduct, or scientific validity—including changes to eligibility criteria, study objectives, sample size or data collection procedures—will be submitted for approval to the relevant research ethics committee and communicated to all participating sites. The trial registry will be updated accordingly.

### Safety considerations

A formal risk assessment using the Imperial College London Research Governance and Integrity Team (RGIT) risk assessment template classified the ViSON study as low risk to participants, equivalent to the usual standard of care. All adverse events pertaining to the study procedures will be reported in accordance with the protocol.

### Informed and voluntary consent

All participants must sign the written consent form prior to breath sampling. Participation in ViSON is voluntary. Participants may withdraw from the study at any time without affecting the standard of care that they receive. On withdrawal, no further breath samples or data will be collected; however, existing breath samples and data will be retained for analysis. Participants will not receive any payments, reimbursement of expenses or any other benefits for participating in the research study.

### Data collection and data monitoring

All study data will be collected according to the Data Protection Act 2018 and in line with the General Data Protection Regulation. The researchers and statisticians have implemented data minimisation. Participant demographics and social history, symptoms and medical history will be recorded in the CRF and ViSON REDCap database. REDCap is a secure online database with access limited to trained and authorised research staff. Source data verification will be conducted through central and on-site monitoring.

Breath samples will be processed according to our standardised laboratory workflow, which ensures full traceability of participants’ samples through the LIMS. Participants’ VOC profiles will be obtained via mass spectrometry during breath sample analysis and recorded alongside their clinical data.

Given the observational study design, a formal Data Monitoring Committee is not required. Study monitoring will focus on adherence to sampling SOPs, completeness of CRFs, data accuracy and timely reporting from participating sites. Central (remote) monitoring will be performed regularly, and periodic (12 monthly) site audits will be conducted after site set up.

### Confidentiality

All data will be pseudonymised at the time of collection, and the re-identification key will be stored electronically within an online password-protected database, with access limited to the local research team of the NHS trusts.

Pseudonymised study data will be transferred to external organisations (LSHTM) for analysis via Imperial College’s approved secure data transfer system. Data Sharing Agreements will be in place ahead of any data sharing.

### Dissemination plan

The results will be presented at national and international conferences. Authorship for any publications will follow the International Committee of Medical Journal Editors (ICMJE) guidance. Links to the publications will be shared in the relevant registries. A lay summary of the results will be developed with the PAG and shared on online websites, social media accounts and through OPA and OOSO charity newsletters.

## Supplementary material

10.1136/bmjopen-2025-112493 online supplemental file 1
