# Supplementary material for: Development of a clinical prediction model using volatile organic compounds as breath biomarkers in squamous oesophageal neoplasms: ViSON, protocol for a multicentre case–control study in UK NHS hospitals
Source: BMJ Open. 2026 Sep 28;16(9):e112493. doi: 10.1136/bmjopen-2025-112493 (PMC13629980; doi:10.1136/bmjopen-2025-112493)
Supplement: Supplementary file 1 [file bmjopen-2025-112493.reviewer_comments.pdf]

PEER REVIEW HISTORY

BMJ Open publishes all reviews undertaken for accepted manuscripts. Reviewers are asked to complete a checklist review form and are provided with free text boxes to elaborate on their assessment. These free text comments are reproduced below.

ARTICLE DETAILS

Title (Provisional)

Development of a clinical prediction model utilising volatile organic compounds as breath biomarkers in squamous oesophageal neoplasms: ViSON, protocol for a multicentre case-control study in UK NHS hospitals

Authors

Sharma, Sameera; Murray, James; Parker, Aaron; Jamel, Sara; Antonowicz , Stefan; Phillips, Adele; Bailey, Elaine; Fox, Lyn; Plant, Christine; Converso, Valerio; Ellis, James K; Mukherjee, Somnath; Kumar, Bhaskar; Lo, Terence; Wahed, Shajahan; Alchawaf, Alia; Moore, Andrew R; Boddy, Alex; Scott-Brown, Martin; Thomas, Betsan; Abdelrahman, Tarig; Pucher, Philip H; El-Sharkawy, Ahmed; Forner, Samantha; Wayman, John; Elshaer, Ahmed; Chan, David; Mcnally, Stephen; Petty, Russell; Patil, Pradeep; Bhuva, Neel; Periyathambi, Jambulingam; Bhatt, Lubna; Vohra, Ravinder; Sharples, Linda; Hanna, George B; ViSON Collaborators, .

VERSION 1 - REVIEW

**Reviewer**	**1**
**Name**	**Saya, Sibel**
**Affiliation**	**The University of Melbourne, Department of General Practice**
**Date**	**05-Feb-2026**
**COI**	**None**

Thank you for the opportunity to review this protocol, I read it with interest. The protocol is generally well written and easy to read, and the study design seems appropriate for the level of evidence that is currently available for the test in question. I do have some thoughts and comments for the authors' consideration, some small comments on phrasing and flow etc, and some on the study design and potential sources of bias, and therefore what sorts of studies should be considered next.

Methodological queries

I have some concerns that selection bias could be an issue in this study. Is there any sequential identification or approach by the direct care team or researchers? The methods

state that the direct care team identify eligible participants, which could be a comprehensive identification or there could certainly be some inadvertent cherry-picking. This should be outlined in the Source of Bias section and the Participant Selection section.

I am also a bit unclear about whether this is a one-gate or two-gate enrolment design. Are individuals enrolled then it is determined whether they are a case or control? I.e., are cases and controls sampled from the same sampling frame? OR are they sourced from different clinics where it is already clear if they are a case or control? It is difficult to determine what the underlying cohort is (and therefore the cohort of people this model will be applicable to), as the manuscript stands now. If it is a two-gate design, more detail on the sources of cases and controls is needed.

It would also be important to state whether the breast test always happens before the gold standard test (if the gold standard test happens after enrolment), and also whether the breast test analysis occurs blinded to case/control status.

These papers may help.

Rutjes AW, Reitsma JB, Vandenbroucke JP, Glas AS, Bossuyt PM. Case-control and twogate designs in diagnostic accuracy studies. Clin Chem. 2005;51(8):1335-1341. doi:10.1373/clinchem.2005.048595

doi:10.7326/M18-1377

Sample size – I am not quite clear what the attrition is accounting for? Is this for loss to follow-up between consent and sample collection and/or the case report form? So the sample size target remains 246 in each group with complete data and 259 per group consented? I think rephrasing of this sentence will make this clearer.

Statistical methods

What is the 'screening step' that will identify the 'top' 50 VOCs? How will these be dealt with in the model if they are highly correlated?

The authors rightly point out that their case-control design artificially sets the disease prevalence at 50%, therefore further work is needed. I am not sure, however, that simple recalibration is sufficient. I would suggest that the next step is a one-gate study in the intended population is required. Perhaps that is what was being suggested in the Narrow validation study, but that was not evident in how it is currently presented.

The Future work section would benefit from a framework that outlines the methodological steps required to develop and test a diagnostic risk prediction algorithm and how the proposed studies align to provide the evidence required for clinical use and eventual impact.

https://doi.org/10.1186/s12885-019-5746-6

Minor comments

Introduction

Diagnosis and survival section – the last sentence of the first paragraph could use rephrasing, I'm not sure it currently makes sense.

Diagnosis and survival section, 3rd paragraph – would be useful to have a sentence on %s of OSCCs diagnoses at stage 0-I, II-III and IV, to aid the logical flow of the argument.

Literature review: breath biomarkers in OSCC – "However, they are limited by geography (East Asia), lack of methodological (sampling and analysis) standardisation and robust power calculation." Within both sets of brackets in this sentence, some more description would be useful. I'm not sure what 'sampling and analysis' meanings – would it be useful to add these shortcomings to Table 1?

Proposed solution section – Please provide the statistic from Ref 14 that shows how this test is 'universally acceptable'

VERSION 1 - AUTHOR RESPONSE

REVIEWER COMMENTS - Ms. Sibel Saya, The University of Melbourne

Comment 1: Methodological query - I have some concerns that selection bias could be an issue in this study. Is there any sequential identification or approach by the direct care team or researchers? The methods state that the direct care team identify eligible participants, which could be a comprehensive identification or there could certainly be some inadvertent cherry-picking. This should be outlined in the Source of Bias section and the Participant Selection section.

Response: Thank you for raising these points. Consecutive sampling of all eligible participants during the study period will be performed to minimise the risk of selection bias. This has now been reflected in the 'Source of bias and mitigation strategies' and 'Participant selection' sections.

Source of bias and mitigation strategies -

Page 12; Lines 225-226 - Deleted

Page 12; Lines 227-232 added - *Selection bias will be minimised through consecutive sampling of all eligible participants during the study period. Participants will be identified systematically by members of the direct care team via upper GI multidisciplinary team (MDT) meetings, upper GI endoscopy lists, and outpatient clinic lists. OSCC cases will be identified primarily through MDT meetings, while control participants will be recruited from upper GI endoscopy and outpatient clinic lists to ensure symptom-matched selection.*

Participant selection -

Page 14; Lines 266-272 - Deleted

Page 14; Lines 273-280 added - *Between December 2023 and November 2026, all eligible participants will be identified consecutively by members of the direct care team at participating NHS sites via the upper GI MDT meetings (OSCC) and the upper GI endoscopy lists, or outpatient clinic lists (controls). All eligible participants will be approached regarding participation in the ViSON study. If interested, a Participant Information Sheet (PIS) will be shared, and participants will be given as much time as is feasible to make an informed decision on whether to take part in the study. Where possible, the recruitment visits will be aligned with planned hospital appointments, investigations, or procedures.*

Comment 2: I am also a bit unclear about whether this is a one-gate or two-gate enrolment design. Are individuals enrolled then it is determined whether they are a case or control? I.e., are cases and controls sampled from the same sampling frame? OR are they sourced from different clinics where it is already clear if they are a case or control? It is difficult to determine what the underlying cohort is (and therefore the cohort of people this model will be applicable to), as the manuscript stands now. If it is a two-gate design, more detail on the sources of cases and controls is needed.

Response: The study represents a two-gate case-control design. Cancer cases are recruited from patients proven to have OSCC on histopathology, while control cases are recruited from patients undergoing endoscopy either routinely or through the 'suspected cancer' pathway.

We have revised the 'Study design' section.

Page 10; Lines 181-183 added **-** *...which adopts a two-gate design. Cancer cases will be recruited from patients with histopathologically confirmed OSCC, while controls will be recruited from patients undergoing endoscopy either routinely or via the 'suspected cancer' pathway.*

Comment 3: It would also be important to state whether the breath test always happens before the gold standard test (if the gold standard test happens after enrolment), and whether the breath test analysis occurs blinded to case/control status. These papers may

help - Rutjes AW, Reitsma JB, Vandenbroucke JP, Glas AS, Bossuyt PM. Case-control and two gate designs in diagnostic accuracy studies. Clin Chem. 2005;51(8):1335-1341. doi:10.1373/clinchem.2005.048595, doi:10.7326/M18-1377

Response: For participants in the OSCC group, the breath test will be performed after the reference standard test (upper GI endoscopy) and histopathological confirmation. For control participants, the breath test may be performed either before or after the reference standard test. All breath samples will be pseudonymised at the point of collection. Subsequent handling and analysis will be conducted with the analytical and statistical teams blinded to the outcome of the reference standard test, and therefore to case–control status.

We have made changes to the 'Source of bias and mitigation strategies' and 'Breath collection and storage' sections.

Source of bias and mitigation strategies -

Page 12; Lines 237-239 added - *All breath samples will be pseudonymised at the point of collection. Subsequent handling and analysis will be conducted with the analytical and statistical teams blinded to the outcome of the reference standard test, and therefore to case–control status.*

Breath collection and storage -

Page 14; Lines 282-285 added - *For participants in the OSCC group, the breath test will be performed after the reference standard test and histopathological confirmation. For control participants, the breath test may be performed either before or after the reference standard test. All breath samples will be pseudonymised at the point of collection.*

Comment 4: Sample size – I am not quite clear what the attrition is accounting for? Is this for loss to follow-up between consent and sample collection and/or the case report form? So, the sample size target remains 246 in each group with complete data and 259 per group consented? I think rephrasing of this sentence will make this clearer.

Response: We have clarified that the anticipated 5% attrition accounts for losses due to ineligibility following consent and sample exclusion during quality control procedures. The 'Sample size calculation' section has been amended to reflect this.

Pages 12-13; Lines 250-252 added - *…to accommodate 5% sample loss. This includes losses due to ineligibility following consent and sample exclusion during quality control procedures (e.g. subthreshold detection of internal standards).*

Comment 5: Statistical methods - What is the 'screening step' that will identify the 'top' 50 VOCs? How will these be dealt with in the model if they are highly correlated?

Response: The screening step refers to an initial dimension-reduction process in which volatile organic compounds (VOCs) with minimal signal are excluded prior to model development. Specifically, moderated t-tests with false discovery rate (FDR) correction are used to rank VOCs based on their association with OSCC status. Approximately the top 50 VOCs (or those meeting a pre-specified FDR threshold) are then taken forward for model development. This approach is commonly used in high-dimensional 'omics analyses, and we have clarified this in the manuscript.

With respect to correlated predictors, elastic net regression will be used for model development. This approach combines L1 (LASSO) and L2 (ridge) penalties, enabling both variable selection and effective handling of collinearity. In the presence of correlated VOCs, elastic net tends to retain groups of correlated variables rather than arbitrarily selecting a single predictor.

We have amended the 'Statistical analysis' section.

Page 16; Lines 333-335 added - *This applies moderated t-statistics with empirical Bayes variance shrinkage and false discovery rate correction to rank VOCs by their univariable association with OSCC status. (19)*

Reference 19 - Dunkler D, Sánchez-Cabo F, Heinze G. Statistical Analysis Principles for Omics Data. Methods in Molecular Biology. 2011;719:113–31. doi:10.1007/978-1-61779-027-0_5/FIGURES/2_5 PubMed PMID: 21370081.

Comment 6: The authors rightly point out that their case-control design artificially sets the disease prevalence at 50%, therefore further work is needed. I am not sure, however, that simple recalibration is sufficient. I would suggest that the next step is a one-gate study in the

intended population is required. Perhaps that is what was being suggested in the Narrow validation study, but that was not evident in how it is currently presented.

Response: Thank you for this important comment and we agree that a one-gate study in the intended clinical population is required to adequately assess real-world performance of the CPM. We have clarified the 'Future Work' section to reflect a staged approach, including initial external validation in a similar population using a two-gate design, followed by a prospective one-gate study in patients referred for upper GI endoscopy, and subsequent international validation to assess generalisability.

We have amended the 'Future work' section. Please see the response to comment 3 from the editor.

Comment 7: The Future work section would benefit from a framework that outlines the methodological steps required to develop and test a diagnostic risk prediction algorithm and how the proposed studies align to provide the evidence required for clinical use and eventual impact. https://doi.org/10.1186/s12885-019-5746-6

Response: The 'Future Work' section has been revised to reflect a staged evaluation approach. Please see the response to comment 3 from the editors.

MINOR COMMENTS

Comment 1: Introduction; Diagnosis and survival section – the last sentence of the first paragraph could use rephrasing, I'm not sure it currently makes sense.

Response: We have modified the 'Diagnosis and survival' section to reflect these changes.

Page 6; Lines 122-123 changed - *...focusses on alarm symptoms and older age, potentially limiting its ability to detect OSCC at an early stage.*

Page 6; Line 124-125 amended to ...and diagnosed via the NICE NG12 'suspected cancer' pathway, ...

Comment 2: Diagnosis and survival section, 3rd paragraph – would be useful to have a sentence on %s of OSCCs diagnoses at stage 0-I, II-III and IV, to aid the logical flow of the argument.

Response: Thank you, we have now added the percentages.

Page 6; Lines 128-129 added - *In the UK, the current incidence of OSCC by stage is 3% (stages 0-I), 25% (stage II), 39% (stage III) and 33% (stage IV).*

Page 6-7; Lines 130-131 changed - *Currently, due to advanced stage at diagnosis, fewer than 40%...*

Comment 3: Literature review: breath biomarkers in OSCC – "However, they are limited by geography (East Asia), lack of methodological (sampling and analysis) standardisation and robust power calculation." Within both sets of brackets in this sentence, some more description would be useful. I'm not sure what 'sampling and analysis' meanings – would it be useful to add these shortcomings to Table 1?

Response: We have revised the sentence to give a detailed justification of limitations. A separate column on 'Study limitations' has been added to Table 1.

Page 7; lines 144-146 – Deleted

Page 7; Lines 147-154 added - *As highlighted in the individual study review, the current evidence base is limited by predominantly case–control designs with non-representative control populations, leading to potential selection and spectrum bias. Studies are also largely conducted in East Asian populations, limiting generalisability, and are often small with limited use of formal power calculations. There is further variability in breath sampling protocols, and substantial heterogeneity in analytical approaches, including the use of both targeted and untargeted analyses and a range of platforms (e.g. GC–MS, PTR-MS, HPPI-TOF-MS, and SPME-based methods), which may affect comparability and reproducibility. (8-13)*

Table 1 moved to Supplementary materials due to large size and *added a column addressing study limitations.*

Comment 4: Proposed solution section – Please provide the statistic from Ref 14 that shows how this test is 'universally acceptable'

Response: The MAGIC study recruited participants from a wide age range (18-90 years), with balanced sex distribution, and diverse ethnic backgrounds. More than 99% participants reported the breath test as easy to perform and were similarly willing to repeat the test in the future.

Page 9; line 162-163 - ...*and proven to be highly acceptable (>99%) in a demographically and clinically representative population.*

Comment 5: If you have selected 'Yes' above, please provide details of any competing interests - Not applicable

---

VERSION 2 - REVIEW

**Reviewer**	**1**
**Name**	**Saya, Sibel**
**Affiliation**	**The University of Melbourne, Department of General Practice**
**Date**	**19-Jun-2026**
**COI**	

Thank you to the authors for considering my comments and revising their manuscript. I have two very small comments and suggested changes.

In the Introduction, I would like the information from the Magic study that "More than 99% participants reported the breath test as easy to perform and were similarly willing to repeat the test in the future." This makes the statement in the Introduction much more credible to the reader!

Is it possible for a recruited control to become a case? I imagine so, as it is stated that the reference gold standard test could occur before or after the breath test? I think a statement about that process, if it possible would be useful.

VERSION 2 - AUTHOR RESPONSE

REVIEWER COMMENTS - Ms. Sibel Saya, The University of Melbourne

Comment 1: In the Introduction, I would like the information from the Magic study that "More than 99% participants reported the breath test as easy to perform and were similarly willing to repeat the test in the future." This makes the statement in the Introduction much more credible to the reader!

Response: Thank you. This line has been included in the 'Proposed solution: Breath test' section on page 8, lines 154-158.

Comment 2: Is it possible for a recruited control to become a case? I imagine so, as it is stated that the reference gold standard test could occur before or after the breath test? I think a statement about that process, if it is possible would be useful.

Response: Thank you for raising this point. Yes, in the rare event that this occurs, the participant will be moved to the OSCC group. We have now included a paragraph in the 'ViSON study procedures: Breath collection and storage' section on page 13, lines 270-273 to clarify this process.
