# Supplementary material for: Development of a clinical prediction model using volatile organic compounds as breath biomarkers in squamous oesophageal neoplasms: ViSON, protocol for a multicentre case–control study in UK NHS hospitals
Source: BMJ Open. 2026 Sep 28;16(9):e112493. doi: 10.1136/bmjopen-2025-112493 (PMC13629980; doi:10.1136/bmjopen-2025-112493)
Supplement: Supplementary file 2 [file bmjopen-16-9-s001.docx]

| **Author, year of publication** | **Country** | **Study Design** | **Patient cohort** | | **Stage of OSCC** | **Analytical platform** | **VOCs** | **Sensitivity**  **(%)** | **Specificity**  **(%)** | **AUC** | **Study limitations** | **Ref** |
| --- | --- | --- | --- | --- | --- | --- | --- | --- | --- | --- | --- | --- |
|  |  |  | **Cancers** | **Controls** |  |  |  |  |  |  |  |  |
| Zou, 2016 | China | Case-control | N=29 (OAC + OSCC) | N=57 | ND | PTR-MS - Untargeted | Dimethyl sulfide, thioethanol, Phenol, 1, 3-cycloheptadiene, Dimethylsulfone, 2-methyl-1, 3-diazine, 1-methylene-2-cyclohexene, Ethylbenzene, P-xylene, O-xylene, M-xylene, Benzaldehyde, Acetaldehyde and Ethylene oxide | 86.2 | 89.5 | 0.943 | Single-centre, spectrum bias (healthy controls), small sample size, no sample size justification, no external validation | [10] |
| Yang, 2023 | China | Case-control | N=63  (OSCC) | N=138  (113 HC &  25 CAG IM) | I & II  (N= 58)  III & IV  (N=8) | GC-MS - Untargeted | ND | 82.54 | 92.03 | 0.951 | Single-centre, spectrum bias (non-representative controls), no sample size justification, limited external validation | [11] |
| Chen, 2024 | China | Case-control | N=12 OSCC | N=18 | III & IV | TFME + GC-MS - Targeted | -Butanal  -1,2,3-trimethylbenzene  -Decanal | 83  77.8  50 | 83  100  91.7 | 0.893  0.888  0.731 | Single-centre, pilot study with small sample size, no sample size justification, no external validation | [12] |
| Matsumori, 2022 | Japan | Case-control | N=17 (OSCC) | N=9 | III & IV | SPME-GC-MS - Untargeted | Acetonitrile, acetic acid, acetone, and 2-butanone | ND | ND | 0.93 | Single-centre, spectrum bias (control selection unclear), small sample size, no sample size justification, no external validation | [13] |
| Huang, 2021 | China | Case-control | N=216 (203 OSCC & 13 OAC) | N=459 | I to IV | HPPI-TOF-MS - Untargeted | ND | 97.55 | 86.13 | 0.97 | Single-centre, no sample size justification, limited external validation | [14] |
| Sasaki, 2021 | Japan | Case-control | N=100  (64 OSCC  14 HPSCC &  22 Both) | N=275 | I | AERoChrome (GC) - Targeted | Acetaldehyde, A/E ratio | ND | ND | ND | **Single-centre,** single biomarker study, spectrum bias, no sample size justification, no external validation | [15] |

*Table 1: Summary of breath VOC studies in oesophageal squamous cell carcinoma; OAC, oesophageal adenocarcinoma; OSCC, oesophageal squamous cell carcinoma; HPSCC, hypopharyngeal squamous cell carcinoma; HC, healthy controls; CAG IM, chronic atrophic gastritis with intestinal metaplasia; ND, no data; PTR-MS, proton transfer reaction mass spectrometry; GC, gas chromatography; HPPI-TOF-MS, high pressure photon ionization time of flight mass spectrometry; SPME-GC-MS, solid phase microextraction gas chromatography mass spectrometry; TFME, thin film microextraction; VOCs, volatile organic compounds; A/E, Acetaldehyde/Ethanol ratio; AUC, Area under receiver operating curve and Ref, Reference.*

# **Participating NHS Trusts**

(i) Imperial College Healthcare NHS Trust, (ii) Norfolk and Norwich University Hospitals NHS Foundation Trust, (iii) Hull University Teaching Hospitals NHS Trust, (iv) The Newcastle Upon Tyne Hospitals NHS Foundation Trust, (v) The Clatterbridge Cancer Centre NHS Foundation Trust, (vi) Liverpool University Hospitals NHS Foundation Trust, (vii) University Hospitals of Leicester NHS Foundation Trust, (viii) University Hospitals Coventry and Warwickshire NHS Trust, (ix) Oxford University Hospitals NHS Foundation Trust, (x) Velindre University NHS Trust, (xi) Cardiff and Vale University Health Board, (xii) Portsmouth University Hospitals NHS Trust, (xiii) University Hospitals of Derby and Burton NHS Foundation Trust, (xiv) Maidstone and Turnbridge Wells NHS Trust, (xv) North Cumbria Integrated Care NHS Foundation Trust, (xvi) Bradford Teaching Hospitals NHS Foundation Trust, (xvii) University Hospital Plymouth NHS Trust, (xviii) NHS Highlands, (xix) NHS Tayside, (xx) East and North Hertfordshire NHS Trust, (xxi) Bedfordshire Hospitals NHS Foundation Trust, (xxii) The Christie NHS Foundation Trust, and (xxiii) Nottingham University Hospitals NHS Trust.
